# Supplementary material for: Evolution and Functional Characteristics of the Novel elovl8 That Play Pivotal Roles in Fatty Acid Biosynthesis
Source: Genes (Basel). 2021 Aug 23;12(8):1287. doi: 10.3390/genes12081287 (PMC8392482; doi:10.3390/genes12081287)
Supplement: Supplementary file 1 [file genes-12-01287-s001.zip › genes-1290660-supplementary.pdf]

Title: Evolution and Functional Characteristics of the Novel Elovl8 Which Play Pivotal Roles in Fatty Acid Biosynthesis

Shouxiang Sun[1,2†], Yumei Wang[1†], Pei-Tian Goh[3], Mónica Lopes-Marques[4], L. Filipe C. Castro[4,5], Óscar Monroig[6], Meng-Kiat Kuah[3], Xiaojuan Cao[1,2], Alexander Chong Shu-Chien[3*], Jian Gao[1,2*]

1. Key Lab of Freshwater Animal Breeding, College of Fisheries, Ministry of Agriculture, Huazhong Agricultural University, Wuhan 430070, China; ssx0707@webmail.hzau.edu.cn (S.S.); YumeiWang@webmail.hzau.edu.cn (Y.W.); caoxiaojuan@mail.hzau.edu.cn (X.C.)

2 Ministry of Education/Hubei Provincial Engineering Laboratory for Pond Aquaculture, Engineering Research Center of Green development for Conventional Aquatic Biological Industry in the Yangtze River Economic Belt, College of Fisheries, Huazhong Agricultural University, Wuhan 430070, China

3 Center for Chemical Biology, Universiti Sains Malaysia, Penang 11800, Malaysia;

gohpeitian@gmail.com (P.-T.G.); kuahmk@gmail.com (M.-K.K.)

4 CIIMAR/CIMAR—Interdisciplinary Center of Marine and Environmental Research, University of Porto, Porto 4099002, Portugal; monicaslm@hotmail.com (M.L.-M.); filipe.castro@ciimar.up.pt (L.F.C.C.)

5 Department of Biology, Portugal/FCUP—Faculty of Sciences, University of Porto, Porto 4099002, Por-tugal

6 Instituto de Acuicultura Torre de la Sal (IATS-CSIC), Ribera de Cabanes, Castellon 12595, Spain; oscar.monroig@csic.es

[†] These two authors contributed equally to this work and should be considered co-first authors

*Corresponding author

Email address: gaojian@mail.hzau.edu.cn (J. Gao)

Postal address: No.1 Shizishan Stress, Hongshan District, Wuhan 430070, Hubei Province, China

Email address: alex@usm.my (Shu-Chien AC)

Postal address: Center for Chemical Biology, Universiti Sains Malaysia, 11800, Minden, Penang, Malaysia.

Figure S1-S5

Table S1

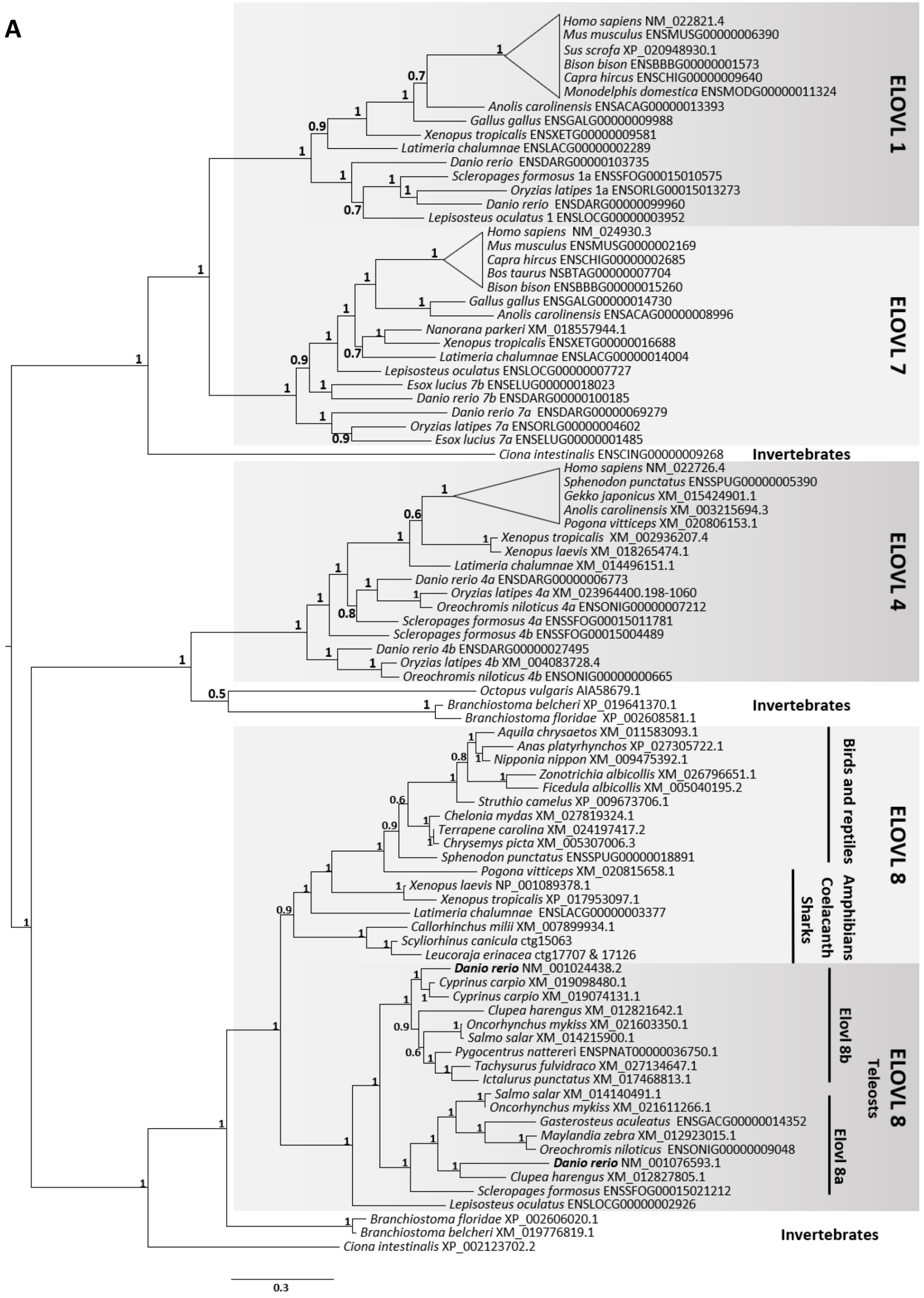
A
ELOVL 1
Homo sapiens NM_022821.4
Mus musculus ENSMUSG00000006390
Sus scrofa XP_020948930.1
Bison bison ENSBBBG00000001573
Capra hircus ENSCHIG00000009640
Monodelphis domestica ENSMODG00000011324
Anolis carolinensis ENSACAG00000013393
Gallus gallus ENSGALG00000009988
Xenopus tropicalis ENSXETG00000009581
Latimeria chalumnae ENSLACG00000002289
Danio rerio ENSDARG00000103735
Scleropages formosus 1a ENSSFOG00015010575
Oryzias latipes 1a ENSORLG00015013273
Danio rerio ENSDARG00000099960
Lepisosteus oculatus 1 ENSLOCG00000003952
ELOVL 7
Homo sapiens NM_024930.3
Mus musculus ENSMUSG0000002169
Capra hircus ENSCHIG00000002685
Bos taurus NSBTAG00000007704
Bison bison ENSBBBG00000015260
Gallus gallus ENSGALG00000014730
Anolis carolinensis ENSACAG00000008996
Nanorana parkeri XM_018557944.1
Xenopus tropicalis ENSXETG00000016688
Latimeria chalumnae ENSLACG00000014004
Lepisosteus oculatus ENSLOCG00000007727
Esox lucius 7b ENSELUG00000018023
Danio rerio 7b ENSDARG00000100185
Danio rerio 7a ENSDARG00000069279
Oryzias latipes 7a ENSORLG00000004602
Esox lucius 7a ENSELUG00000001485
Ciona intestinalis ENSCING00000009268
Invertebrates
ELOVL 4
Homo sapiens NM_022726.4
Sphenodon punctatus ENSSPUG00000005390
Gekko japonicus XM_015424901.1
Anolis carolinensis XM_003215694.3
Pogona vitticeps XM_020806153.1
Xenopus tropicalis XM_002936207.4
Xenopus laevis XM_018265474.1
Latimeria chalumnae XM_014496151.1
Danio rerio 4a ENSDARG00000006773
Oryzias latipes 4a XM_023964400.198-1060
Oreochromis niloticus 4a ENSONIG00000007212
Scleropages formosus 4a ENSSFOG00015011781
Scleropages formosus 4b ENSSFOG00015004489
Danio rerio 4b ENSDARG00000027495
Oryzias latipes 4b XM_004083728.4
Oreochromis niloticus 4b ENSONIG00000000665
Octopus vulgaris AIA58679.1
Branchiostoma belcheri XP_019641370.1
Branchiostoma floridae XP_002608581.1
Invertebrates
ELOVL 8
Birds and reptiles
Aquila chrysaetos XM_011583093.1
Anas platyrhynchos XP_027305722.1
Nipponia nippon XM_009475392.1
Zonotrichia albicollis XM_026796651.1
Ficedula albicollis XM_005040195.2
Struthio camelus XP_009673706.1
Chelonia mydas XM_027819324.1
Terrapene carolina XM_024197417.2
Chrysemys picta XM_005307006.3
Sphenodon punctatus ENSSPUG00000018891
Pogona vitticeps XM_020815658.1
Amphibians
Coelacanth
Sharks
Xenopus laevis NP_001089378.1
Xenopus tropicalis XP_017953097.1
Latimeria chalumnae ENSLACG00000003377
Callorhinchus milii XM_007899934.1
Scyliorhinus canicula ctg15063
Leucoraja erinacea ctg17707 & 17126
ELOVL 8
Teleosts
Elovl 8b
Danio rerio NM_001024438.2
Cyprinus carpio XM_019098480.1
Cyprinus carpio XM_019074131.1
Clupea harengus XM_012821642.1
Oncorhynchus mykiss XM_021603350.1
Salmo salar XM_014215900.1
Pygocentrus nattereri ENSPNAT00000036750.1
Tachysurus fulvidraco XM_027134647.1
Ictalurus punctatus XM_017468813.1
Elovl 8a
Salmo salar XM_014140491.1
Oncorhynchus mykiss XM_021611266.1
Gasterosteus aculeatus ENSGACG00000014352
Maylandia zebra XM_012923015.1
Oreochromis niloticus ENSONIG00000009048
Danio rerio NM_001076593.1
Clupea harengus XM_012827805.1
Scleropages formosus ENSSFOG00015021212
Lepisosteus oculatus ENSLOCG00000002926
Branchiostoma floridae XP_002606020.1
Branchiostoma belcheri XM_019776819.1
Invertebrates
Ciona intestinalis XP_002123702.2
0.3

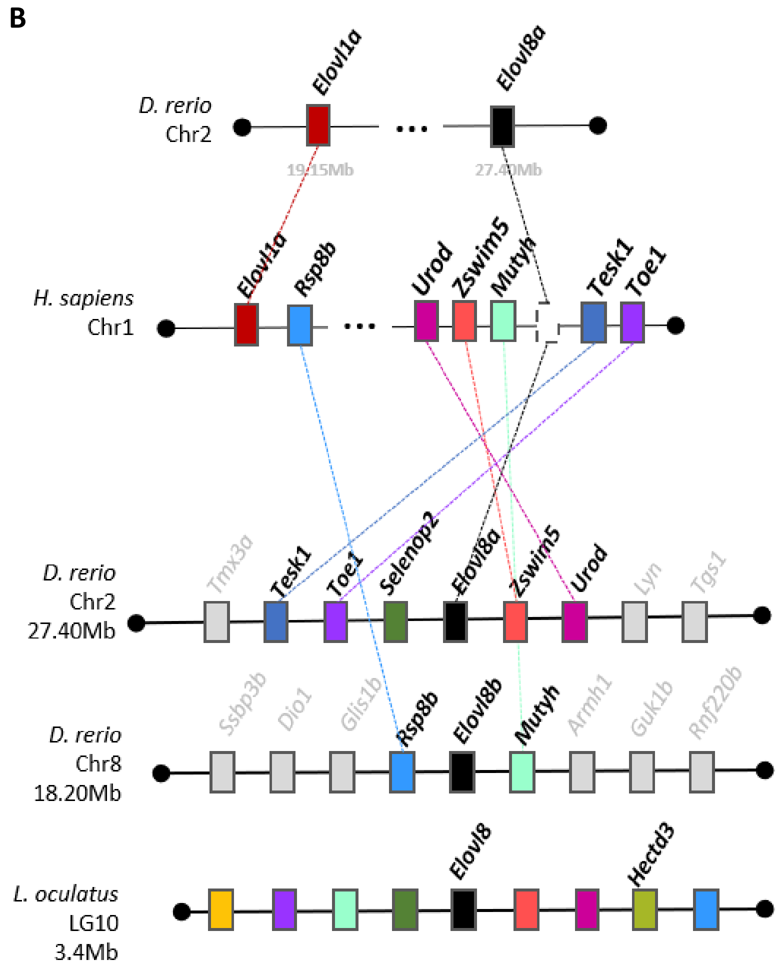

Figure S1. Phylogenetic analysis and synteny maps of Elovl8.

(A) Phylogenetic analysis of Elovl1, Elovl7 Elovl4 and Elovl8 sequences, values at node correspondent to posterior probabilities provided by aBayes. Tree was rooted at midpoint. (B) Syntenic location of the Elovl8 genes in several species; Elovl8 gene is represented by black box; dotted black box in human represents a pseudogene; color code of the remaining boxes is conserved corresponding to the same gene identified in several species. Genes identified in grey show no conservation with the remaining genes.

Elovl: elongation of very long-chain fatty acid protein.

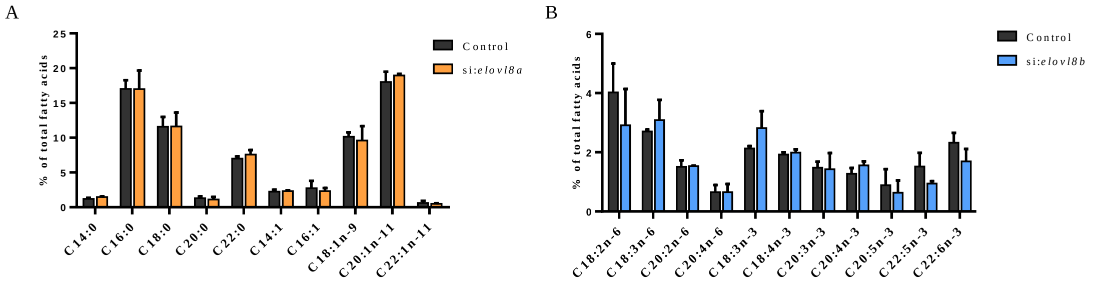

Figure S2. Effects of *elovl8a* and *elovl8b* knockdown on liver fatty acid composition. (A) SFA and MUFA composition of control and si:*elovl8a* treated ZFL cells. (B) PUFA composition of control and si:*elovl8b* treated ZFL cells.

elovl, elongation of very long-chain fatty acid protein; SFA, saturated fatty acid; MUFA, mon-ounsaturated fatty acids; PUFA, polyunsaturated fatty acids.

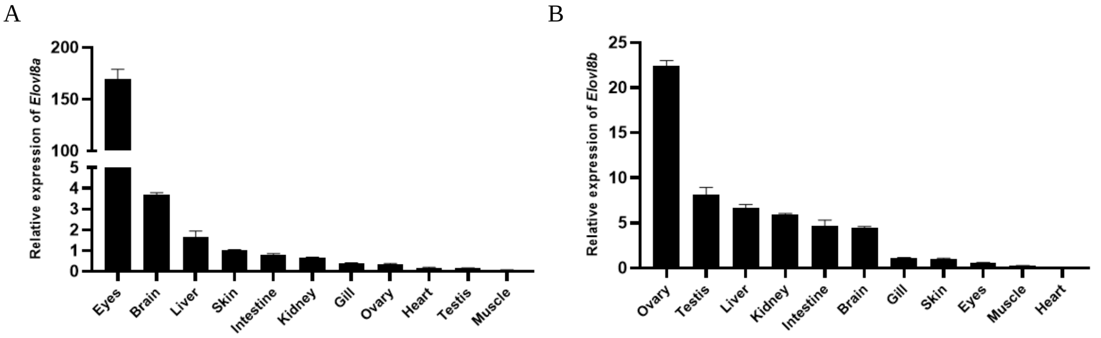

Figure S3. Relative mRNA expression levels of *elovl8a* (A) and *elovl8b* (B) in different tissues of wild-type zebrafish. Data were expressed as mean ± SD of three biological replicates.

A

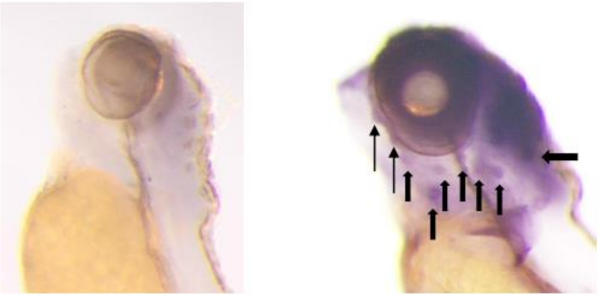

B.

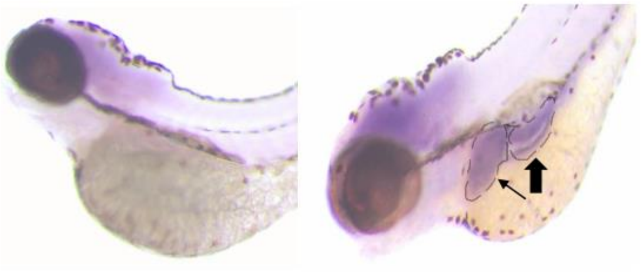

Figure S4. Expression of *elovl8a* (A) and *elovl8b* (B) in 96hpf zebrafish. Images on left showed fish labeled with sense riboprobe as negative control. For *elovl8a,* expression was prominent in eyes (arrow) and head neuromasts (bold arrow). As for *elovl8b*, expression was detected in the developing liver (arrow) and intestine (bold arrow). *elovl*, elongastion of very long-chain fatty acid protein; hpf, hours post-fertilization.

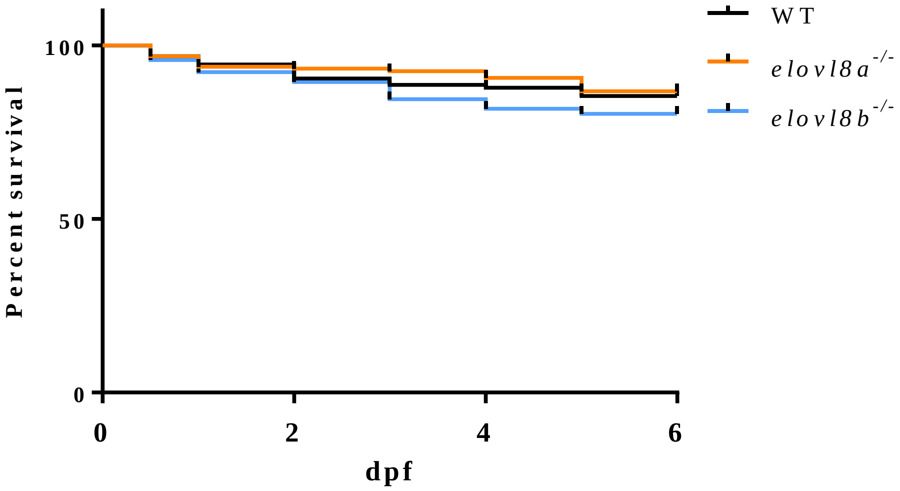

Figure S5. Survival rates of the wild-type (WT), *elovl8a*$^{-/-}$ and *elovl8b*$^{-/-}$early embryo stage. *elovl*, elongation of very long-chain fatty acid protein; dpf, days post-fertilization.

Table S1. The primers of PCR used in this study.

Target gene primers	Primer sequences (5´-3´)	Accession numbers
Construction of knockout models		
elovl8a-F	GGTCTGAATTGCTTTCTTC	ID: 767653
elovl8a-R	ATGTTTTTATTTGTCTATGGTC	
elovl8b-F	ATAGATCTGTGTACTTCTGCA	ID: 554145
elovl8b-R	CAGTCCTTTTAGGTTCACTGGT	
qPCR		
elovl2-F	GTTTTCAGCTGTCCCGTA	NM_001040362.1
elovl2-R	ATTGGAATGACTGTGTTTAGG	
elovl5-F	CCAAGGACAGGACGAAGC	NM_200453.2
elovl5-R	CAGTGTGCAAACGTGTAAGGA	
elovl4a-F	GTCATTCTTCGGGGCTCACA	NM_200796.1
elovl4a-R	CCGATCAGACACCAGTGCAT	
elovl4b-F	CTTTGATTGGCTATGCCGTTAC	NM_199972.1
elovl4b-R	CGTGCTTTCCTTTTCCTTTCTT	
elovl5-F	CCAAGGACAGGACGAAGC	NM_200453.2
elovl5-R	CAGTGTGCAAACGTGTAAGGA	
elovl7a-F	TGTATGGCATCATCTTCCTCCT	NM_199875.1
elovl7a-R	GCAGTCGGCAGAGTAACCT	
elovl7b-F	GCGGTTCTGCTGTATGATGAG	NM_199778.1
elovl7b-R	ACGATGCTGAGGTTGTAGATGA	
elovl8a-F	ACGGAGACAAGAGGACAGATG	NM_001076593.1
elovl8a-R	TGCCAACCAAGAGGAGACTG	
elovl8b-F	AATCCGCATGGCAGAGACT	NM_001024438.2
elovl8b-R	CCAAGATGTGACAAGGAACTCA	
gapdh-F	TCCAGTACGACTCCACCCAT	NM_001115114.1
gapdh-R	TGACTCTCTTTGCACCACCC	
β-actin-F	CACCACCACAGCCGAAAGAG	AF057040.1
β-actin-R	ACCGCAAGATTCCATACCCA	
Primers for in situ hybridization riboprobe synthesis		
elovl8a-F	GAGACAAGAGGACAGATGGATG	
elovl8a-R	CTAGCTTGGTCTTCTTGGAGAG	
elovl8b-F	CCCATGGCTACTAGTCTACTC	
elovl8b-F	AGCTGGACATTTCACCTCTCTC	
elovl8a and *elovl8b* knockdown		
si:*elovl8a*	GGAUGGCUGUUGGUUUAUUTT	
si:*elovl8b*	GGAUCGGACCCAAGCUUAUTT	

Elovl, elongases of very long-chain fatty acids; *gapdh*: glyceraldehyde-3-phosphate dehydrogenase.
